# American Medical Association

**Published:** 1858-06

**Authors:** 


### EDITORIAL AND MISCELLANEOUS.

### American Medical Association.

We had the pleasure of attending the late meeting of the American Medical Association in Washington City, and while our feelings would prompt us to go into very considerable detail, we must content ourselves for the present (in consequence of pressing engagements,) with a bare reference to the many interesting features of the late meeting.

Upon the Physicians of the District of Columbia, too much praise cannot be bestowed, for the elegant and profuse hospitality extended to the members of the Association. Besides the magnificent entertainments in Washington City—the excursion to Mount Vernon, and the unique and delightful dinner at the Pavilion, (upon the Potomac,)—afforded the most conclusive evidence of the elevated, refined and generous character of the profession, whose hospitality seemed to know no bounds. To the President of the United States, Senators Douglas and Toombs, and perhaps others, is also due praise, for their attention to members of the Association.

The number of delegates in attendance was very large, (over 400,) among whom were found many of the most prominent medical men in the United States.

We think the forthcoming volume of the transactions will be unusually interesting and valuable; at least, so far as the *papers* presented, are concerned; for the discussions in the Association we can not say so much; a great deal of the time of the body was consumed in an utterly fruitless way, but even doctors are sometimes fond of speech-making, and, taking the locality into consideration, they ought, perhaps, in this instance to be excused.

One of the most prominent reports before the body, was that of the Special Committee on Medical Education, by the

Chairman, Dr. J. R. Wood, of New York city. This report, we propose to discuss in the next number of this Journal, at least, certain features, which we have no hesitation in asserting, bear the *impress* of *the fixed purpose*, upon the part of certain cliques, to control the Association, and make it the instrument of destruction to certain troublesome competitors.

The whole movement resulting in the *present* excitement upon this subject, we profess to understand, and at the proper time shall most unquestionably hold up to the indignation of the profession, who have no part or lot, no word or interest in the contemptible and selfish juggling and trickery, which certain parties have introduced into the workings of this highly honorable and useful body. Our firm conviction is, in reference to this, the most national meeting of the Association ever held, that the mass of the body had no inclination to consider the subject of medical education, which can never be controlled by the Association, and which has only proved a source of annoyance heretofore. In conclusion, we have to say that if the American Medical Association is to be converted into a "*Court* for the correction of Errors," embracing within its *grasp* this vast Union, it will soon be numbered among the things that were.

